# Biodistribution of biodegradable polymeric nano-carriers loaded with busulphan and designed for multimodal imaging

**DOI:** 10.1186/s12951-016-0239-0

**Published:** 2016-12-19

**Authors:** Heba Asem, Ying Zhao, Fei Ye, Åsa Barrefelt, Manuchehr Abedi-Valugerdi, Ramy El-Sayed, Ibrahim El-Serafi, Khalid M. Abu-Salah, Jörg Hamm, Mamoun Muhammed, Moustapha Hassan

**Affiliations:** 1 Division of Functional Materials (FNM), Department of Materials and Nanophysics, Royal Institute of Technology (KTH), 164 40 Stockholm, Sweden; 2 Division of Experimental Cancer Medicine (ECM), Department of Laboratory Medicine (LABMED), Karolinska Institutet (KI), 141 86 Stockholm, Sweden; 3 Clinical Research Center (KFC), Karolinska University Hospital Huddinge, 141 86 Stockholm, Sweden; 4 Department of Nanomedicine, King Abdullah International Medical Research Center, King Abdulaziz Medical City, PO Box 22490, Riyadh, 11426 Saudi Arabia; 5 PerkinElmer, 68 Elm St., Hopkinton, MA 01748 USA

**Keywords:** Biodegradable polymer, Drug delivery, Magnetic resonance imaging, In vivo fluorescence imaging, Biodistribution, Busulphan, Cancer

## Abstract

**Background:**

Multifunctional nanocarriers for controlled drug delivery, imaging of disease development and follow-up of treatment efficacy are promising novel tools for disease diagnosis and treatment. In the current investigation, we present a multifunctional theranostic nanocarrier system for anticancer drug delivery and molecular imaging. Superparamagnetic iron oxide nanoparticles (SPIONs) as an MRI contrast agent and busulphan as a model for lipophilic antineoplastic drugs were encapsulated into poly (ethylene glycol)-*co*-poly (caprolactone) (PEG-PCL) micelles via the emulsion-evaporation method, and PEG-PCL was labelled with VivoTag 680XL fluorochrome for in vivo fluorescence imaging.

**Results:**

Busulphan entrapment efficiency was 83% while the drug release showed a sustained pattern over 10 h. SPION loaded-PEG-PCL micelles showed contrast enhancement in *T*_2_*-weighted MRI with high *r*_2_* relaxivity. In vitro cellular uptake of PEG-PCL micelles labeled with fluorescein in J774A cells was found to be time-dependent. The maximum uptake was observed after 24 h of incubation. The biodistribution of PEG-PCL micelles functionalized with VivoTag 680XL was investigated in Balb/c mice over 48 h using in vivo fluorescence imaging. The results of real-time live imaging were then confirmed by ex vivo organ imaging and histological examination. Generally, PEG-PCL micelles were highly distributed into the lungs during the first 4 h post intravenous administration, then redistributed and accumulated in liver and spleen until 48 h post administration. No pathological impairment was found in the major organs studied.

**Conclusions:**

Thus, with loaded contrast agent and conjugated fluorochrome, PEG-PCL micelles as biodegradable and biocompatible nanocarriers are efficient multimodal imaging agents, offering high drug loading capacity, and sustained drug release. These might offer high treatment efficacy and real-time tracking of the drug delivery system in vivo, which is crucial for designing of an efficient drug delivery system.

**Electronic supplementary material:**

The online version of this article (doi:10.1186/s12951-016-0239-0) contains supplementary material, which is available to authorized users.

## Background

Nanoparticles offer great potential for various biomedical applications such as drug delivery [1–3], diagnostics [4, 5] and bioimaging [6, 7]. With rapidly developing techniques of nanomaterials, it has become possible to create advanced multifunctional drug delivery carriers. These all-in-one carriers offer the possibility of fulfilling several therapeutic needs simultaneously, such as delivery of therapeutic cargo, real-time imaging, targeting and controlled release [8–10]. Biodegradable polymers are attractive biomaterials that can be utilized for nanomedicine [11–14]. Properties such as controlled and sustained drug delivery, improved drug pharmacokinetics, reduced side effects and biodegradability make these materials ideal carrier systems for a variety of functional agents [15]. Amphiphilic biodegradable polymers can self-assemble and form micellar architecture in aqueous media [16]. Therefore, an important benefit of these polymeric micelles in medical application [17] is that they have the ability to load and deliver the contrast agents or drugs to targeting sites where imaging and therapy should take place [18].

Imaging moieties are often incorporated into drug delivery carriers for tracking and evaluation purposes. Among them, the inorganic contrast agent superparamagnetic iron oxide nanoparticles (SPIONs) is widely used in magnetic resonance imaging (MRI) [13, 19] owing to their ability of shortening the spin–spin relaxation time and significantly increasing the imaging contrast [20]. However, un-functionalized SPIONs tend to aggregate and form clusters after intravenous injection due to van der Waals interactions between the particles [13] and, hence, the aggregated particles are rapidly eliminated by macrophages in the mononuclear phagocyte system (MPS) [21]. This undesired behavior can be avoided by using amphiphilic block copolymers as carriers to protect SPIONs from the surrounding environment. Several studies have previously reported SPIONs loaded into PEG-PCL copolymers as contrast agents for MRI [22, 23]. The amphiphilic block copolymers can form micellar nanoparticles with a hydrophobic core and a hydrophilic shell. The hydrophobic core allows entrapment of agents with low aqueous dispersion, such as hydrophobic SPIONs and lipophilic drugs, while the hydrophilic shell can render aqueous dispersion and enhance colloidal stability of the polymeric nanoparticles.

Optical imaging is based on non-invasive detection of fluorescence or luminescence, enabling time course data acquisition and minimizing inter-individual variation. By adding a fluorescent tag to existing contrast agents used in other imaging modalities, dual or multimodal imaging can be realized, such as optical imaging/MRI, optical imaging/CT and optical imaging/ultrasound. In our previous study, air-filled polyvinyl alcohol microbubbles (PVA MBs) were labeled with a near infrared (NIR) fluorophore, VivoTag 680XL, and proved to be a useful contrast agent for both ultrasound imaging and fluorescence imaging [24]. In vivo optical imaging of fluorescence probe labeled contrast agents in small animals facilitates pre-clinical biodistribution and kinetic studies without the need for radio isotopes, and the in vivo behavior can ultimately be verified by fluorescence microscopy.

Busulphan is an alkylating agent that is efficient in low doses for the treatment of chronic myeloid leukemia [25] and in high doses as conditioning regimen prior to stem cell transplantation (SCT) [26]. Busulphan interacts chemically with the DNA to terminate its replication and induce DNA damage. High-dose regimen of busulphan was reported to cause sinusoidal obstructive syndrome (SOS) known previously as veno-occlusive disease (VOD) [27]. Furthermore, high variation in bioavailability in combination with inter-individual variation in PK increases the need for an intravenous (iv) formulation. However, the poor water solubility of busulphan has limited the possibility for an optimal delivery system. These limitations could be overcome by encapsulating busulphan into polymeric nanocarriers to achieve its therapeutic potential.

In the current study, poly (ethylene glycol)-*co*-poly (caprolactone) (PEG-PCL) micelles have been prepared as a multifunctional carrier (theranostic) for encapsulating busulphan for drug delivery, SPIONs for MR imaging and fluorescence probe VivoTag 680XL for optical fluorescence imaging. The copolymer was synthesized by the ring opening polymerization technique using tin (II) 2-ethylhexanoate as a catalyst. The prepared copolymer was characterized by Fourier transform infrared spectroscopy (FTIR), thermogravimetric analysis (TGA), differential scanning calorimetry (DSC), and proton nuclear magnetic resonance (^1^H-NMR). The multifunctional micelles were prepared by the single oil-in-water (O/W) emulsion-solvent evaporation method which incorporated theranostic agents, i.e., SPIONs and busulphan. The phantom study of SPION-loaded PEG-PCL micelles shows a high *r*_2_* relaxivity under *T*_2_*-weighted MRI. The PEG-PCL nanocarriers with a payload of busulphan as well as SPIONs exhibit high drug entrapment efficiency and slow drug release in PBS solution at 37 °C and pH 7.4. The cytotoxicity of the PEG-PCL micelles has been evaluated in the HL60 cell line. Furthermore, biodistribution of VivoTag 680XL labeled-PEG-PCL micelles has been studied by in vivo fluorescence imaging. And their distribution in different organs was later confirmed by histological analysis. These results suggest that the multifunctional PEG-PCL micelles designed and developed in this study are useful tools for drug delivery, and to facilitate drug delivery with imaging guided approaches.

## Methods

### Reagents and chemicals

ε-Caprolactone monomer (ε-CL, 99%), tin (II) 2-ethylhexanoate, polyvinyl alcohol (PVA), *N*-hydroxysuccinimide (NHS), 1-ethyl-3-(3-dimethylaminopropyl) carbodiimide (EDC), 5-carboxyfluorescein, (3-aminopropyl) trimethoxysilane (APTMS), MTT reagent (3-(4, 5-dimethylthiazol-2-yl)-2, 5-diphenyltetrazolium bromide), and poly (ethylene glycol) monomethyl ether (PEG) (molecular weight approximately 5 kDa) were purchased from Sigma-Aldrich Chemical Co., (Munich, Germany). PEG was dried under vacuum at 60 °C for 48 h before use. VivoTag^®^ 680XL (λ_ex_ = 665 ± 5 nm, λ_em_ = 688 ± 5 nm) was purchased from PerkinElmer Co., (Boston, MA, USA). Dichloromethane (DCM) and all solvents were provided by Sigma-Aldrich Chemical Co., (Munich, Germany).

### Synthesis and characterization of PEG-PCL copolymer

Amphiphilic PEG-PCL copolymer was synthesized by ring opening polymerization of ε-CL monomer using macroinitiator of PEG and catalyst of tin (II) 2-ethylhexanoate as reported previously [28, 29]. The prepared polymer was characterized by a Bruker AM 400 proton nuclear magnetic resonance (^1^H-NMR) (Bruker Co., Billerica, USA) at 400 MHz using deuterated chloroform (CDCl_3_) as solvent. The solvent signal was used as an internal standard. Fourier transform infrared (FTIR) spectroscopy was performed using a Thermo Scientific Nicolet iS10 spectrometer (Thermo Fisher Scientific Co., Kungens Kurva, Sweden) in the attenuated total reflection (ATR) mode with a ZnSe crystal. Gel permeation chromatography (GPC) with dimethylformamide (DMF) as mobile phase was used to determine the molecular weight and polydispersity index (PDI) of polymers. The analyses were performed on an EcoSEC HLC-8320 GPC system (TOSOH Co., Tokyo, Japan) equipped with an EcoSEC RI detector and three columns (PSS PFG 5 µm; Microguard, 100, and 300 Å) (PSS GmbH, Frankfurt, Germany). The calibration curve was established using mono-dispersed poly (methyl methacrylate) standards. The thermal behavior including melting temperature of the PEG-PCL copolymer was measured using differential scanning calorimetry DSCQ2000 (TA Instruments, MA, USA) at a constant heating rate (10 °C/min) ranging from 25 to 100 °C. Thermogravimetric analysis was performed on a TGA-Q500 (TA Instruments, MA, USA) to detect the changes of polymer sample weight with regard to temperature increase to 700 °C at a constant heating rate of 10 °C/min under nitrogen as the purging gas.

### Preparation and characterization of SPION-loaded PEG-PCL micelles

Monodispersed SPIONs were synthesized using the thermal decomposition method according to our previous work [30]. Briefly, an iron-oleate complex synthesized from the reaction of sodium oleate and FeCl_3_·6H_2_O was decomposed into SPIONs in a solvent of octyl ether at approximately 297 °C. SPION-PEG-PCL micelles were prepared by the O/W emulsion solvent evaporation technique. Briefly, an appropriate amount of PEG-PCL polymer was dissolved in DCM and SPIONs in the same solvent were added. The organic solution was mixed with aqueous poly (vinyl alcohol) (PVA) solution (1:10 oil to water ratio) under probe type sonication for 15 min to form an emulsion. The resulting brownish emulsion was stirred overnight to evaporate organic solvent at room temperature. The obtained SPION-PEG-PCL micelles were then washed three times using de-ionized (DI) water (15 MΩ cm at 25 °C).

The morphologies of SPION-PEG-PCL micelles were examined by field emission transmission electron microscopy (FE-TEM) JEM-2100 (JEOL Ltd., Tokyo, Japan) operating at an accelerating voltage of 200 kV. Several drops of the suspended micelles were placed on a carbon film copper grid and positively stained using a 2% aqueous solution of phosphotungstic acid (H_3_PW_12_O_40_). The hydrodynamic diameter of the SPION-PEG-PCL micelles was measured using dynamic light scattering (DLS) Delsa™Nano particle size analyzer (Beckman Coulter, Brea, CA, USA). The concentration of iron was measured by Thermo Scientific iCAP 6500 inductively coupled plasma atomic emission spectroscopy (ICP-AES) (Thermo Fisher Scientific Co., Kungens Kurva, Sweden). The optical absorbance and fluorescence intensity of fluorescein-PEG-PCL micelles were measured by Lambda 900 UV–Vis–NIR spectrometer (Perkin Elmer, Waltham, MA, USA) and LS 55 Fluorescence spectrometer (Perkin Elmer, Waltham, MA, USA), respectively.

### In vitro drug release

To study the busulphan release from SPION-PEG-PCL micelles, 30 mg busulphan and 50 mg PEG-PCL were dissolved in DCM with SPION solution to form an organic phase. The organic phase was then emulsified with aqueous PVA solution under probe type sonication. After evaporation of the organic solvent, drug-loaded micelles were recovered by centrifugation at 7800 rpm for 20 min and washed using DI water (15 MΩ.cm) several times to remove physical absorbed or unloaded drug. The washed busulphan-loaded SPION-PEG-PCL micelles were redispersed in 3 ml PBS and placed in a dialysis tube with a molecular weight cutoff (MWCO) of 10 kDa to dialyze against PBS (pH 7.4) solution at 37 ± 0.4 °C under continuous shaking at 80 rpm (Multitron shaker, INFORS HT, Bottmingen, Switzerland). Using this method, the drug is allowed to be released through the porous polymer surface and permeated into dialysis media through pores on the dialysis membrane due to the concentration difference. At predetermined intervals, 5 ml aliquots were withdrawn and replaced with fresh medium adjusted to 37 °C. The concentration of released busulphan as well as the drug retained in the dialysis bag after the release period was measured by gas chromatography (SCION 436-GC; Bruker, Billerica, MA, USA) with electron capture detector (ECD) according to a method reported previously by Hassan et al. [31]. Entrapment efficiency of busulphan in SPION-PEG-PCL micelles was calculated as [(amount of drug released from the micelles + residual drug in the dialysis membrane)/(total amount of drug added initially) × 100%].

### In vitro magnetic resonance imaging

MRI phantoms were made of SPION-PEG-PCL micelles (10 ml) with iron concentrations of 0.1, 0.3, 0.5 and 1 mM. Phantoms were prepared by mixing the micelle suspension with agarose (Sigma-Aldrich Chemical Co., Munich, Germany) aqueous solution (3%) which was heated and then cooled down in falcon tubes overnight to form a gel. The phantoms were placed in the extremity coil of a clinical 3T MR scanner (Siemens, Erlangen, Germany) at room temperature and a gradient echo *T*_*2*_ * sequence was applied. The repetition time was 2000 ms and six stepwise increasing echo times (TEs) of 2–17.2 ms was used to obtain the *T*_*2*_ *-weighted images of the phantoms. Regions of interest (ROI) were manually placed on the images. The relaxation time, *T*_*2*_ *, was then calculated as the slope of a semi-log plot of the signal intensity in the ROI versus the TEs. The relaxivity *R*_*2*_ * was calculated as 1/*T*_*2*_ *. All *R*_*2*_ * values for the phantoms were subtracted by *R*_*2*_ * value for the control sample (plain agarose gel). A standard curve was plotted with *R*_*2*_ * (s^−1^, y-axis) versus iron concentration (mM, x-axis).

### In vitro cellular uptake

The fluorescein-labeled PEG-PCL was prepared by a one-step conjugation reaction. In brief, the amino terminated PEG-PCL copolymer was synthesized by adding 2 mmol APTMS mixed with 0.3 mmol PEG-PCL copolymer solution in tetrahydrofuran, and reflux under N_2_ overnight. The copolymer was collected by precipitation in diethyl ether, filtered and dried. The carboxy fluorescein was then conjugated with amino-terminated copolymer via carbodiimide crosslinking using EDC and NHS. The micelles were prepared from fluorescein-PEG-PCL in DCM and emulsified in PVA aqueous solution.

For the cell uptake study, the murine macrophage cell line (J774A, the European Type Tissue Culture Collection) (CAMR, Salisbury, UK) was a gift from Professor Carmen Fernandez (Department of Immunology, Wenner-Gren Institute, Stockholm University, Stockholm, Sweden). J774A cells were cultured in Dulbecco’s modified Eagle medium (DMEM) supplemented with 10% heat-inactivated fetal bovine serum (Invitrogen), penicillin (100 µg/ml) (invitrogen), and streptomycin (100 µg/ml) (Invitrogen) in 50 cm^2^ tissue culture flasks (Costar, Corning, NY, USA). The cultures were maintained at 37 °C under 5% carbon dioxide. J774A cells were cultured in 8-chamber polystyrene vessel tissue culture treated glasses at a density of 5 × 10^5^ cells/chamber at 37 °C for 12 h. Thereafter, the cell culture medium was aspirated from each chamber and substituted with the medium alone (control) or the same medium containing fluorescein-PEG-PCL micelles at concentrations of 1000 μg/ml. Chambers were then incubated at 37 °C for 4 and 24 h under 5% carbon dioxide. The intracellular uptake of micelles was terminated at each time point by aspirating the test samples, removing the chamber and washing the cell monolayers with ice-cold PBS three times. Each slide was then fixed with methanol-acetone (1:1 v/v), followed by examination under fluorescence microscopy. The uptake of fluorescein-PEG-PCL micelles could be visualized by virtue of the intrinsic green fluorescence of fluorescein dye by employing fluorescent microscope Eclipse i80 (Nikon, Tokyo, Japan) at a wavelength of 520 nm.

### In vitro MTT assay

HL60 cells were seeded at a density of 10,000 cells per well in 96-well plate and maintained in Roswell Park memorial institute medium (RPMI 1640) supplemented with 10% heat-inactivated fetal bovine serum, penicillin (100 µl) and streptomycin (100 µl). After incubation with different concentrations of PEG-PCL micelles for 48 h in a humidified incubator (5% carbon dioxide) at 37 °C, MTT reagent was added to each well. The cells were further incubated for 4 h at 37 °C. The solubilizing agent was added to each well and crystals were solubilized by pipetting up and down. The absorbance was measured at 570 nm, and absorbance at 690 nm was used as reference. PBS was used as blank and cells in medium as control. The cell viability percentage was calculated as (cells treated with the micelles/non-treated control cells) × 100%.

### In vivo fluorescence imaging/computed tomography (CT)

Animal studies were approved by the Stockholm Southern Ethical Committee and performed in accordance with Swedish Animal Welfare law. The distribution of fluorescence was observed by an IVIS^®^ Spectrum (PerkinElmer, Waltham, MA, USA). Quantum FX (Perkin Elmer, Waltham, MA, USA) was also used to co-register functional optical signals with anatomical μCT. Balb/C mice (22 ± 2 g) were purchased from Charles River (Charles River Laboratories, Sulzfeld, Germany) and kept for one week in the animal facility to acclimatize before the experiments. The animals had free access to food and water, ad libitum, and were kept in a 12 h light/dark cycle under controlled humidity (55% ± 5%) and temperature (21 °C ± 2 °C). Prior to all experiments, mice were fed for three weeks on a synthetic diet free of unrefined chlorophyll-containing ingredients (alfalfa free, Research diets, Inc., USA) to minimize the fluorescence noise signal from the gastrointestinal tract.

The VivoTag 680XL-labeled PEG-PCL was prepared as follows: a solution of VivoTag 680XL in dimethyl sulfoxide (0.37 mg/ml) was mixed with amino-terminated PEG-PCL copolymer solution under stirring for 1 h. The labeled copolymer was collected by precipitation in cold diethyl ether, and dried at room temperature. The non-conjugated VivoTag 680XL was removed by dialysis (MWCO 10 kDa) against PBS at room temperature. The VivoTag 680XL-labeled PEG-PCL micelles were prepared by the formerly described emulsion solvent evaporation method. A suspension of VivoTag 680XL-labeled PEG-PCL micelles (0.2 ml equivalent to 4 mg/mouse) was intravenously injected into the lateral tail vein of the mice. The mice (n = 3 per time point) were anaesthetized using 2–3% isoflurane (Baxter Medical AB, Kista, Sweden) during the whole imaging procedure. The 2D/3D fluorescence imaging and µCT scans were performed at 1, 4, 24 and 48 h post injection. The Mouse Imaging Shuttle (MIS, 25 mm high, PerkinElmer) was used to transfer the mice from the IVIS Spectrum to the Quantum FX-µCT while maintaining their positions. Mice were firstly imaged by 2D epi-illumination fluorescent imaging in a ventral position. Subsequently, the mice were imaged in the MIS using 3D Fluorescent Imaging Tomography (FLIT) with trans-illumination in a dorsal position. The 3D FLIT imaging sequence was set up and images were acquired at excitation 675 nm and emission 720 nm. The mouse in the MIS was then transferred to the Quantum FX-µCT and subjected to a fast, low dose CT scan with a field of view (FOV) at 60 mm and 17 s scan-time. All images were generated using the Living Image^®^ 4.3.1 software (PerkinElmer, Waltham, MA, USA).

### Necropsy and histology

The mice (n = 3) were sacrificed at pre-determined time points immediately after the imaging procedure, and histological analysis of lungs, liver, spleen and kidneys was performed using phase contrast and fluorescence microscopy. To verify the observations from the in vivo live imaging, the organs were removed from the mice, fixated in paraformaldehyde (4%) for 24 h, then transferred to ethanol (70%), routinely processed and embedded in paraffin. Later, tissue sections (4 µm) were mounted on super frost glass slides. Slides were routinely stained with 4′,6-diamidino-2-phenylindole (DAPI, 300 nM) to produce nuclear counter stain for fluorescence microscopic evaluation. H&E staining was performed according to the manufacturer’s instructions. Six sections were examined for each sample.

## Results

### Synthesis and characterization of PEG-PCL copolymer

**Fig. 1:**
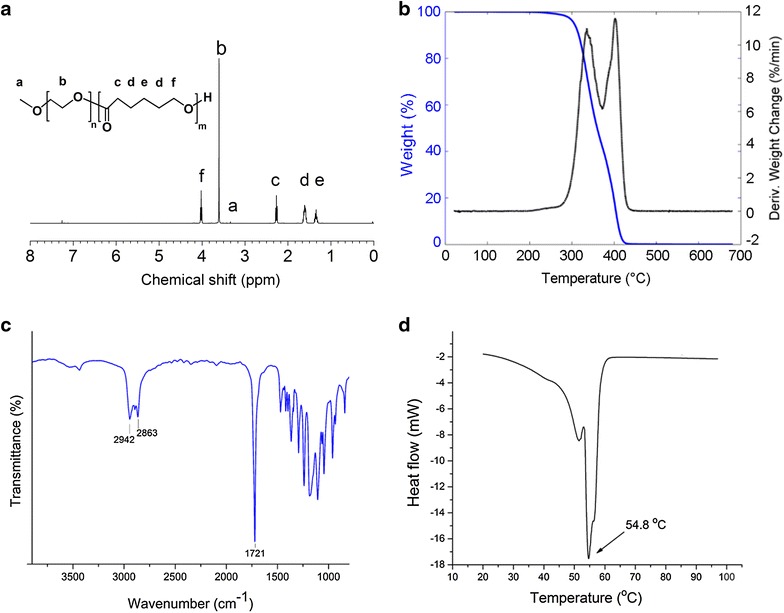
Characterization of PEG-PCL copolymer. **a** Proton nuclear magnetic resonance, **b** Thermogravimetric analysis of PEG-PCL copolymer spectra showing the diblock structure of copolymer. **c** The FTIR spectra of PEG-PCL copolymer, where the observed strong and sharp absorption band appearing at 1721 cm^−1^ is characteristic of the stretching vibration of the ester carbonyl group (C=O) of PCL. The (C–H) stretching bands in PEG and PCL are vibrating at 2863 and 2942 cm^−1^, respectively; **d** Thermal property of PEG-PCL diblock copolymer. The melting temperature of the copolymer was measured by differential scanning calorimetry (DSC) and a bimodal melting peak was observed at 54.8 °C

The biodegradable polymer, poly (ε-caprolactone) (PCL), was polymerized in the presence of PEG using the ring opening polymerization method. The prepared PEG-PCL copolymer shows a narrow molecular weight distribution with an average molecular weight of 30.63 kDa and a polydispersity index of 1.4. The structural analysis of the prepared PEG-PCL copolymer is shown in Additional file 1: Figure S1. The ^1^H-NMR spectrum of PEG-PCL copolymer (Fig. 1a) exhibits a sharp singlet peak at 3.60 ppm, which is attributed to the methylene protons of the PEG blocks unit in the PEG-PCL copolymer. It is clearly seen from the spectrum that there are two equally intense triplet peaks at 2.26 and 4.01 ppm, assigned to the methylene protons in the PCL chain. Additionally, successful synthesis of a copolymer was confirmed by thermogravimetric analysis; as shown in Fig. 1b, PEG-PCL was thermally decomposed at two weight loss events. These two stages of copolymer weight loss correspond to the hydrophilic and hydrophobic polymer chains in the PEG-PCL copolymer. The FTIR spectra of the PEG-PCL diblock copolymer has been illustrated in supplementary data (Fig. 1c). A strong sharp absorption band appearing at 1721 cm^−1^ is characteristic of the stretching vibration of the ester carbonyl group (C=O) of PCL. The (C–H) stretching bands in PEG and PCL vibrate at 2863 and 2942 cm^−1^, respectively. The melting temperature of PEG-PCL diblock copolymer was measured by DSC (Fig. 1d), and a bimodal melting peak was observed at 54.8 °C. All these chemical shifts and peaks confirm the chemical structure of the prepared diblock copolymer which is similar to the results of previous studies on synthesized PEG-PCL copolymer [32–34].

### Synthesis and characterization of SPION-loaded PEG-PCL micelles

**Fig. 2:**
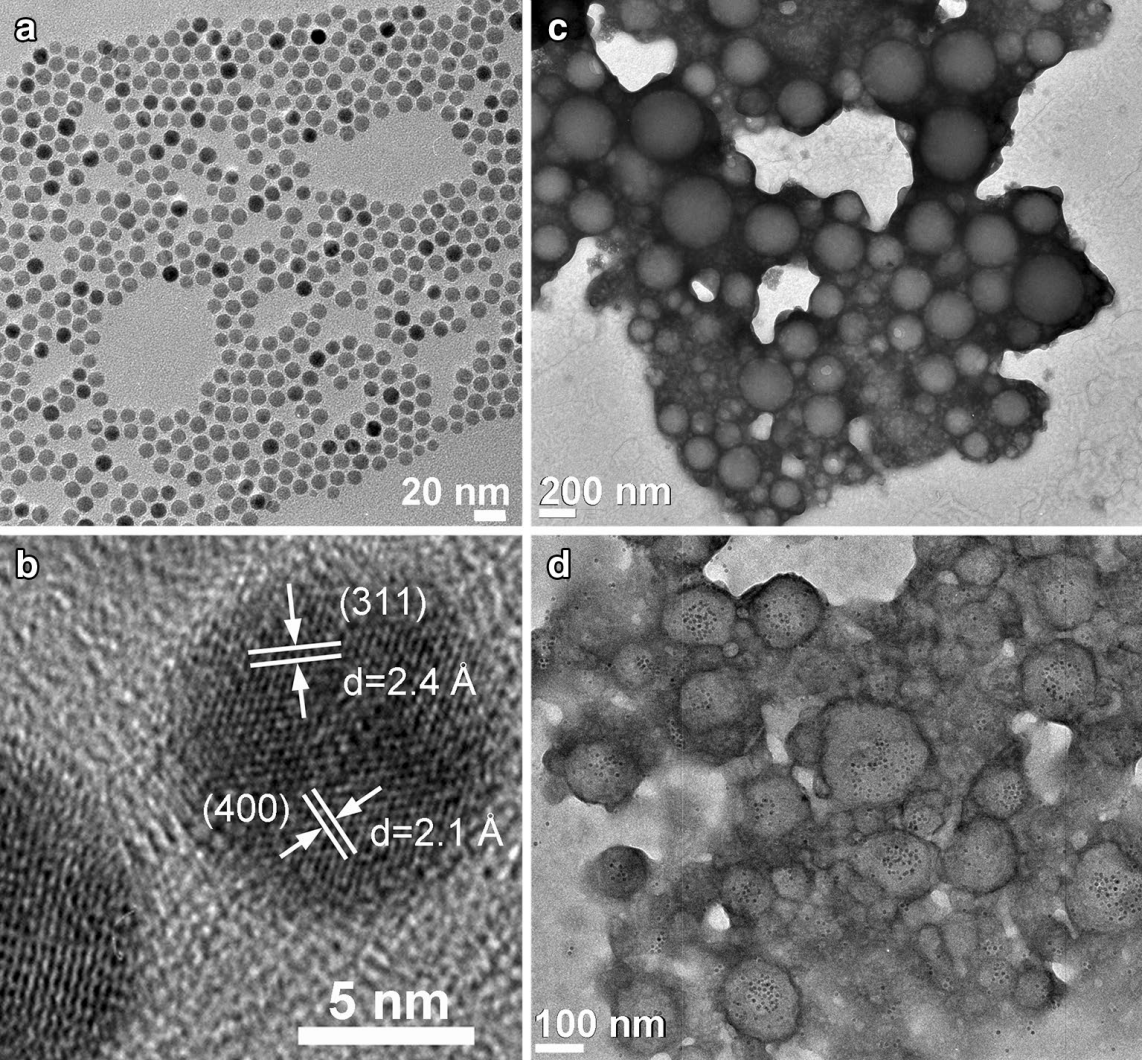
Morphological images of SPIONs, PEG-PCL micelles and SPION-loaded polymer micelles using field emission transmission electron microscopy (FE-TEM). **a** SPIONs, **b** high resolution image of a single SPION, **c** positively stained PEG-PCL micelles, **d** positively stained SPION-PEG-PCL micelles

The micelles were synthesized from the prepared PEG-PCL copolymer by the O/W emulsion solvent evaporation technique. The oil phase containing copolymer and SPIONs was emulsified into an aqueous phase containing PVA as a stabilizing agent. The morphology of SPIONs, PEG-PCL micelles without SPIONs and SPION-loaded polymer micelles was investigated by TEM. Figure 2a shows the uniform size distribution of the SPIONs with average diameter of 10.7 nm (standard deviation σ ~ 8%) as reported in our previous results [30]. A high resolution TEM image (Fig. 2b) shows the single crystal structure of SPION. According to the TEM micrograph of non-loaded PEG-PCL micelles (Fig. 2c), the average diameter is 212 nm (σ ~ 30%). The loading of SPION into PEG-PCL nanocarriers is clearly seen in Fig. 2d. The distribution of the hydrodynamic size of SPION-PEG-PCL micelles is shown in Additional file 1: Figure S1. The surface charge of SPION-PEG-PCL micelles was measured with a negative zeta potential of approximately −2.8 mV at neutral pH.

### In vitro drug release

**Fig. 3:**
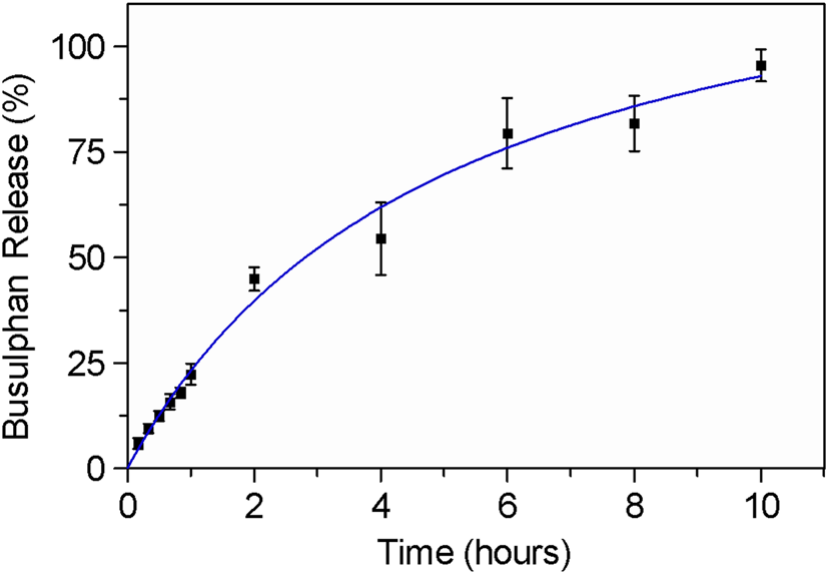
Busulphan release from SPION-PEG-PCL micelles in PBS solution at 37 ± 0.4 °C. Data points represent average values (n = 3) ± SD

Busulphan, a hydrophobic anticancer drug, was entrapped in the hydrophobic core of the PEG-PCL micelles using the O/W emulsion solvent evaporation technique. The entrapment efficiency of busulphan in the PEG-PCL micelles was calculated as 83 ± 2%. The release behavior of busulphan from SPION-PEG-PCL micelles was studied in a PBS dissolution medium at pH 7.4 and 37 ± 0.4 °C. The drug release profile against time is illustrated in Fig. 3. The percentage of busulphan released was calculated by dividing the amount of drug diffused from the dialysis membrane to the release media by the total drug amount loaded into the PEG-PCL nanocarrier. Sustained drug release pattern was observed during 10 h and around 98% of the drug was released.

### In vitro magnetic resonance imaging

**Fig. 4:**
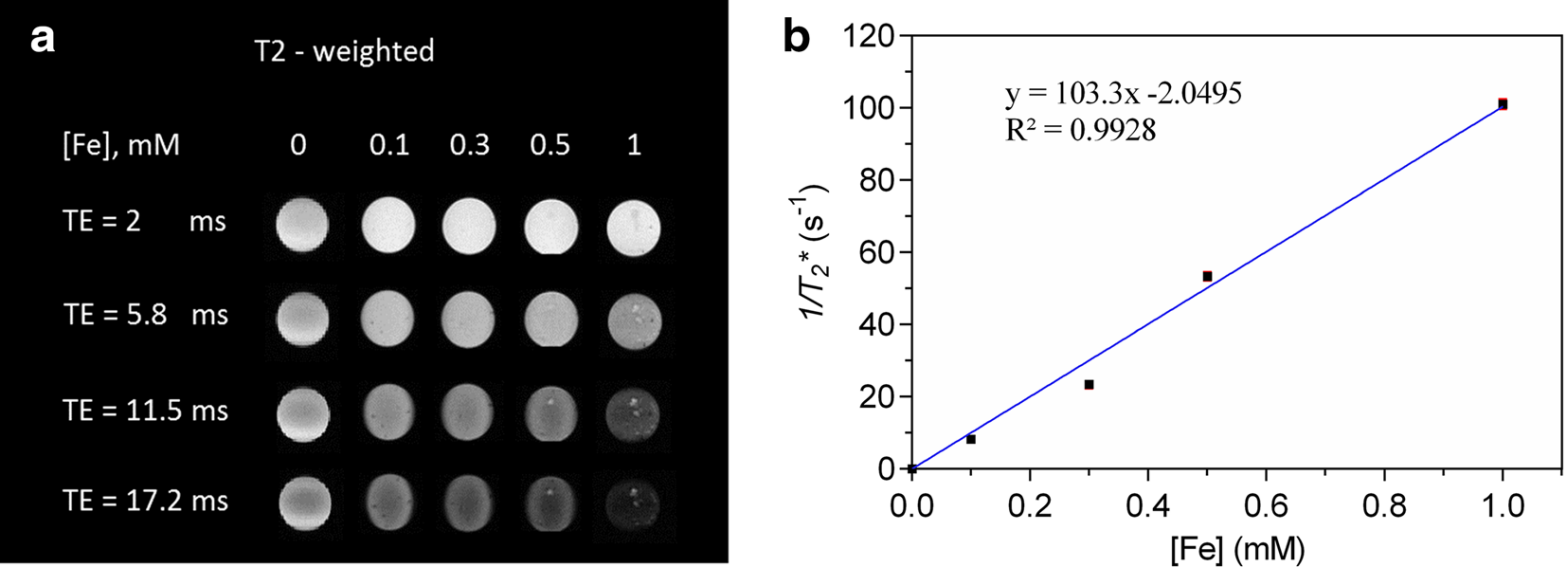
Magnetic resonance imaging (MRI) of SPION-PEG-PCL micelle phantom. **a** *T*_2_^*^-weighted MR phantom images of SPION-PEG-PCL at different TEs (TR = 1200 ms; TE = 2, 5.8, 11.5 and 17.2 ms), **b** Proton transverse relaxation rate (*R*_2_^*^ = 1/*T*_2_^*^) of phantom samples versus iron concentration in mM

The *T*_*2*_ *-weighted MRI was measured on phantoms containing SPION-PEG-PCL micelles with different iron concentrations at different echo times (TEs). The *T*_*2*_ *-weighted MRI of phantoms is shown in Fig. 4a. We found that by increasing the concentration of iron oxide from 0.1 mM to 1.0 mM in the phantoms of SPION-PEG-PCL micelles, the signal intensity of the MRI was decreased. The relaxivity *r*_2*_ for the SPION-PEG-PCL micelles was calculated from the slope of the linear plots of *R*_*2*_ * relaxation rates versus Fe concentration (Fig. 4b), which is 103.3 Fe mM^−1^ s^−1^.

### In vitro cell uptake

**Fig. 5:**
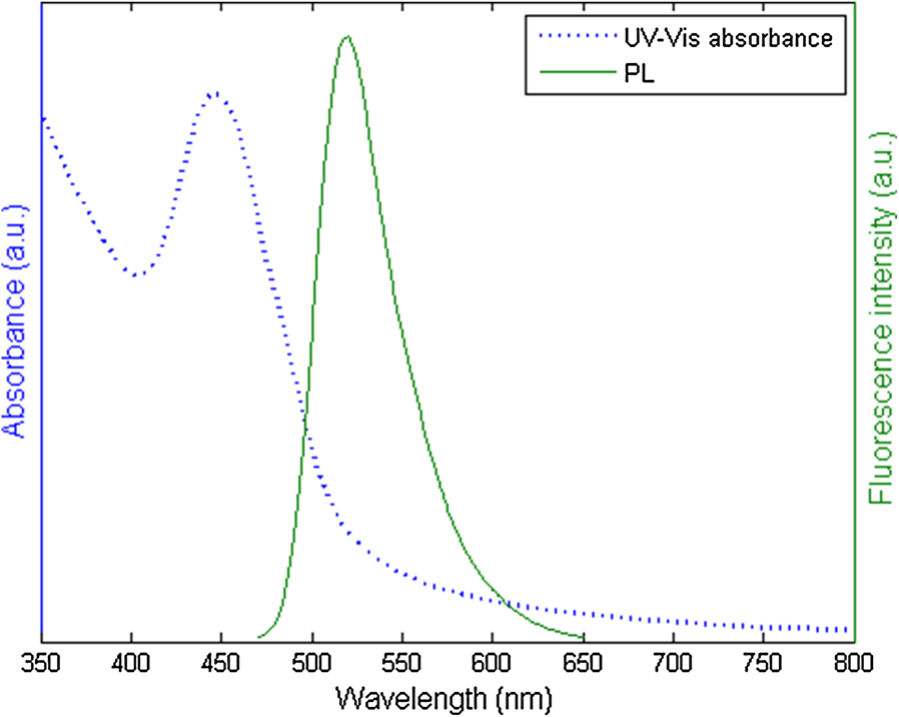
Fluorescence spectrum of fluorescein-labeled PEG-PCL micelles. The maximum absorbance and fluorescence intensity of the fluorescein-PEG-PCL micelles have been observed at 460 and 518 nm, respectively (a.u.: arbitrary unit)

**Fig. 6:**
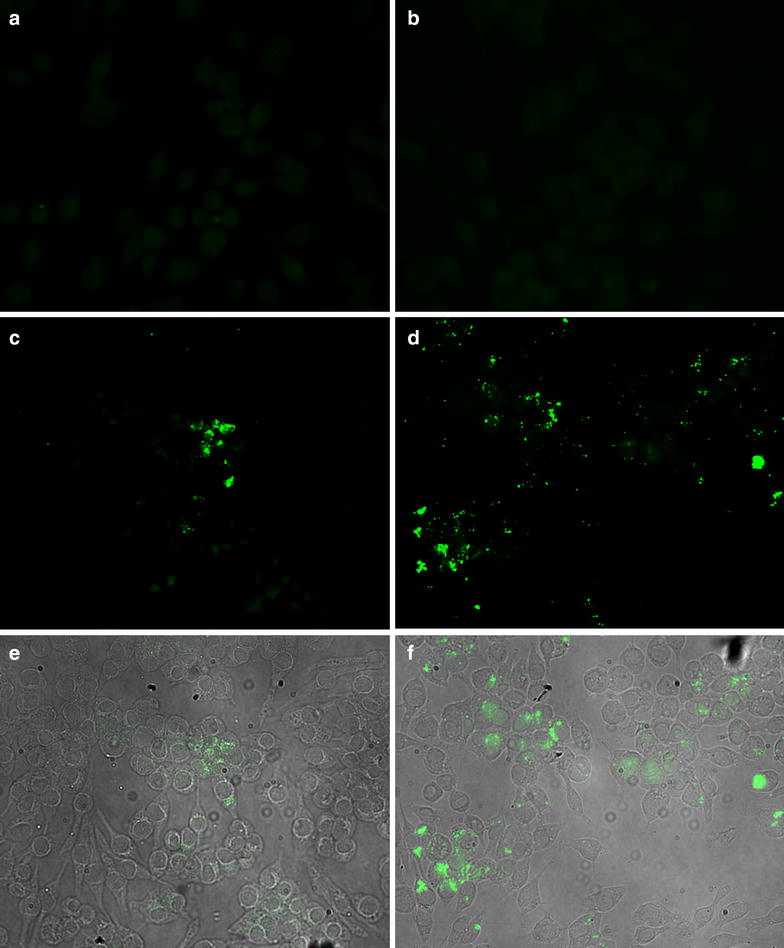
Superimposed fluorescence and light microscopy images of J774A incubated with fluorescein-labeled PEG-PCL micellles. **a**, **b** Non-treated control J774A cells, fluorescence image at 4 and 24 h, respectively, 40×. **c**, **d** Fluorescence images of J774A incubated with nanoparticles for 4 h and 24 h, respectively, 40×. **e**, **f** Overlay images (fluorescence image and light image) of J774A incubated with nanoparticles for 4 and 24 h, respectively, 40×

Cellular uptake and cellular distribution of PEG-PCL micelles were investigated by fluorescence microscopy using fluorescein-labeled PEG-PCL micelles. The carboxy-fluorescein was conjugated with the amino-terminated PEG-PCL copolymer via APTMS linker using carbodiimide chemistry. The conjugation of fluorescein dye was confirmed by measuring the fluorescence intensity of the PEG-PCL micelles after removing the free dye through dialysis. Figure 5 illustrates the UV–VIS absorbance and photoluminescence spectra of the fluorescein-labeled PEG-PCL micelles. The maximum absorbance and fluorescence intensity of the fluorescein-PEG-PCL micelles have been observed at 460 and 518 nm, respectively. Cellular uptake of fluorescein-PEG-PCL NPs was investigated using the murine macrophage cell line J774A. Incubation of micelles with cells was carried out for 4 and 24 h in a humidified incubator (5% carbon dioxide) at 37 °C. Figure 6a and b show the fluorescence imaging of non-treated J774A cells, at 4 and 24 h respectively. The fluorescence images of the uptake of fluorescein-PEG-PCL micelles by the J774A cells at 4 h (Fig. 6c) and at 24 h (Fig. 6d) show initial and maximum fluorescence intensity of the cells treated with fluorescein-PEG-PCL micelles. Internalization of fluorescein-PEG-PCL micelles was observed at 4 and 24 h co-culture as shown in the overlay images of light microscopy and fluorescence microscopy, Fig. 6e and f, respectively. It is worth to mention that micelles were seen in the cell cytosol.

### In vitro cytotoxicity assay

The cytotoxicity of the busulphan free PEG-PCL micelles was evaluated in the HL60 cell line. The cell viability was determined by MTT assay after incubation with PEG-PCL micelles for 48 h and the results are plotted in Additional file 1: Figure S2. Cell viability was calculated as the percentage of living cells treated with micelles to that of the non-treated cells. PEG-PCL micelles do not induce major cytotoxicity at the concentration up to 25 µg/ml, with around 100% cell viability in HL60 cells after PEG-PCL micelles treatment. Cell viability was observed to be 80% at PEG-PCL micelle concentrations of 50 and 100 µg/ml.

### In vivo fluorescence imaging/computed tomography (CT)

**Fig. 7:**
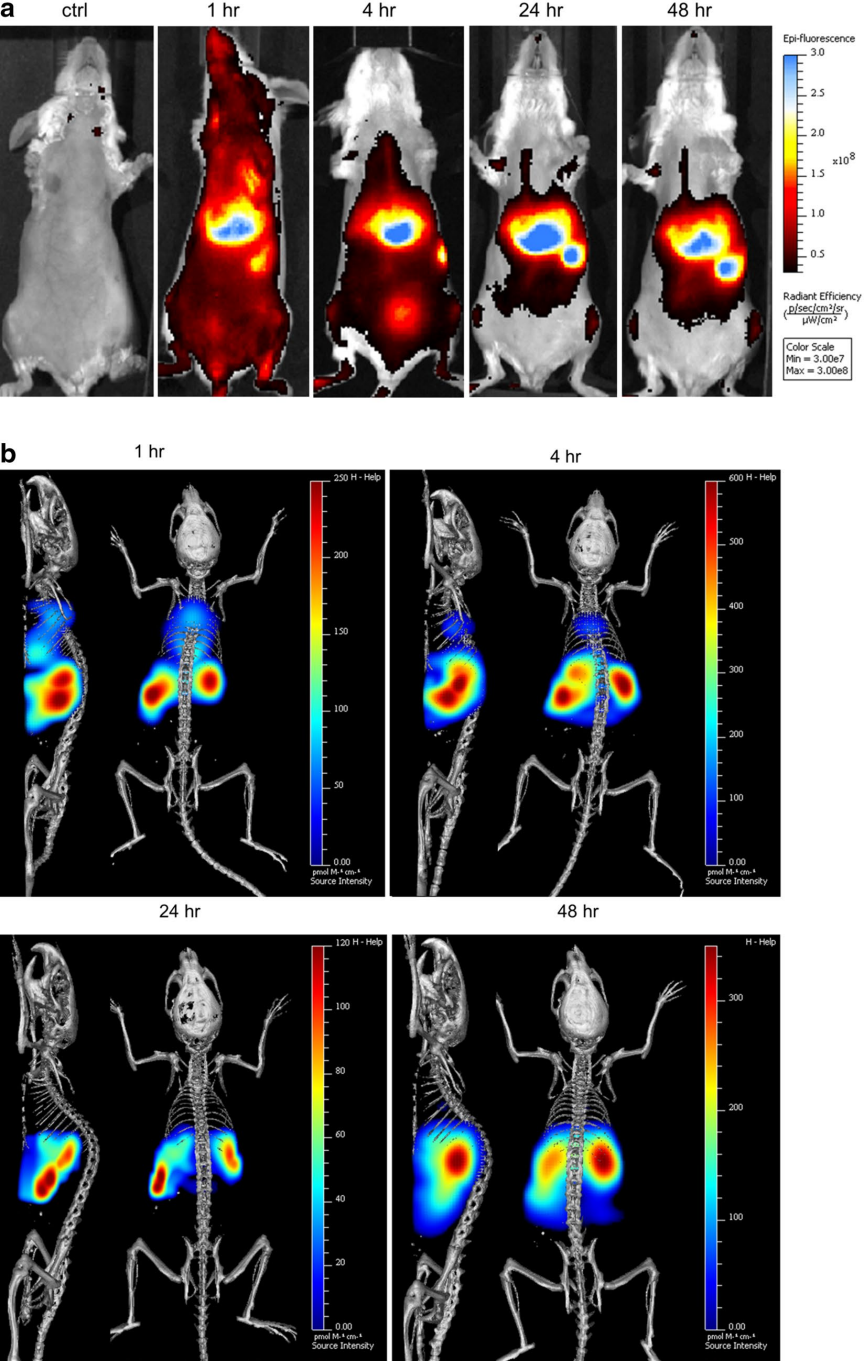
In vivo fluorescence imaging of Balb/c mice after intravenous administration of PEG-PCL micelles labelled with VivoTag 680XL. **a** 2D fluorescence imaging of Balb/c mice at 1, 4, 24 and 48 h after iv administration, *ventral side*. Fluorescence images are overlaid with light photography. Control is a mouse which has not been administered with VivoTag 680XL PEG-PCL micelles. **b** 3D fluorescence imaging-CT imaging co-registration of Balb/c mice at 1, 4, 24 and 48 h after iv administration, *dorsal side* and *left side* as shown. Heat map represents the area with fluorescence signal and *color* represents the intensity

The PEG-PCL micelles were further functionalized for in vivo fluorescence imaging using a near infrared (NIR) fluorescence probe, VivoTag 680XL. VivoTag 680XL is suitable for in vivo fluorescence imaging due to its high light penetration property and low autofluorescence at activation wavelength (Ex/Em 675/720 nm). After iv administration of PEG-PCL micelles, the biodistribution was followed up to 48 h (Fig. 7). Figure 7a shows whole body 2D fluorescence imaging prior to injection and at 1, 4, 24 and 48 h post injection, while Fig. 7b shows whole body 3D fluorescence imaging co-registered with 3D µCT imaging. 3D µCT images provide the anatomical referencing for 3D fluorescence imaging to accurately predict the location the of fluorescence signal as can be seen in the 3D FLIT imaging with trans-illumination in a dorsal position (Additional file 1: Figure S3). Images were acquired at 1, 4, 24 and 48 h post injection. During the first hour, we noticed that micelles distributed primarily into the lungs, liver and spleen. Despite the fact that lungs are the first capillary organ, higher distribution was observed in the spleen and liver 1 h post injection. However, PEG-PCL micelles showed slow clearance over the first 24 h and almost no signal was observed in the lungs at 48 h. At 4 h post injection, the fluorescence intensity was reduced in the lungs, and increased in the spleen and liver. After 24 h, fluorescence intensity in the lungs was further reduced, and signals with equal strength were observed in the liver and spleen. It is worth noticing that micelle distribution was also detected in the femurs. At 48 h, weak fluorescence signal was observed in the lungs and high signal accumulation in the spleen and liver. In general, the fluorescence intensity was decreased with a weak signal still detectable in the femurs.

### Ex vivo fluorescence imaging and histological analysis

**Fig. 8:**
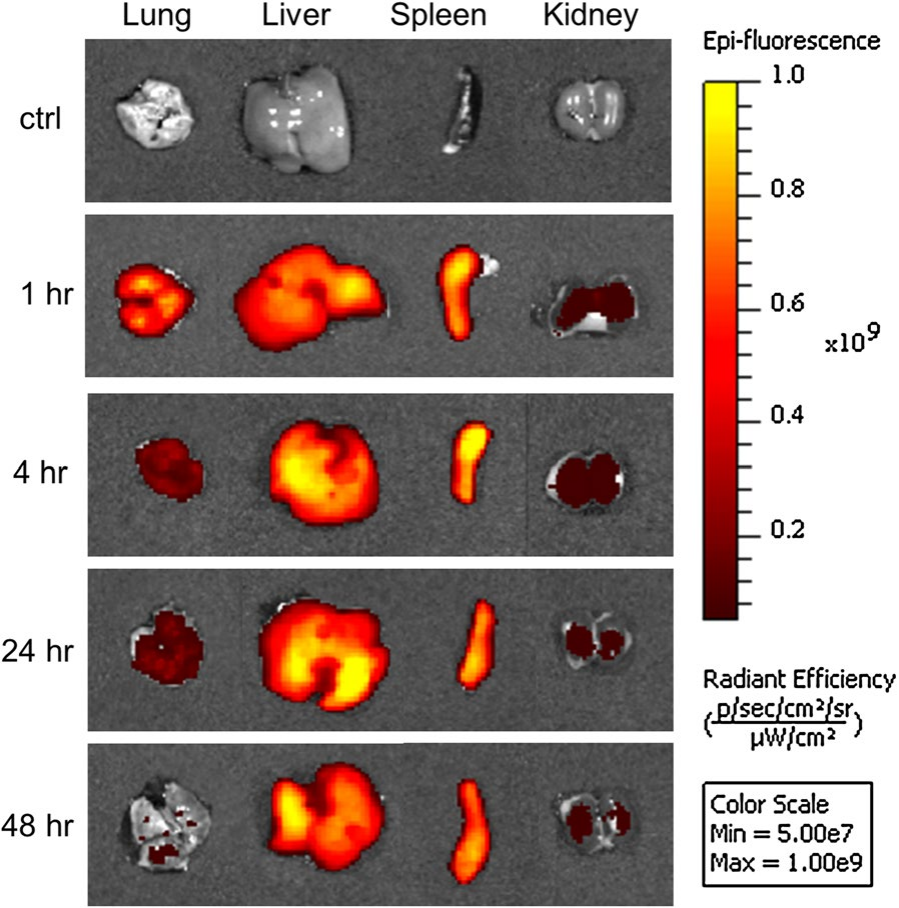
Ex vivo organ fluorescence imaging of lungs, liver, spleen. Images were taken at 1, 4, 24 and 48 h. The signal decreases in the lungs from 1 h until 48 h, and increases in the liver and spleen during the same time. A slight decrease in signal intensity was observed at 48 h in the liver and spleen

Figure 8 shows ex vivo fluorescence images for lungs, liver, spleen and kidneys removed from the sacrificed mice at 1, 4, 24 and 48 h respectively. The ex vivo organ fluorescence images are in agreement with findings from in vivo fluorescence imaging. PEG-PCL micelles were mainly accumulated in the liver and spleen after systemic administration. Until 48 h post injection, the liver and spleen still showed strong fluorescence intensity. A high amount of micelles were captured in the lungs 1 h after iv injection, then quickly cleared from the lungs (4 and 24 h). A low fluorescence signal was generally observed in the kidneys throughout the experiment.

**Fig. 9:**
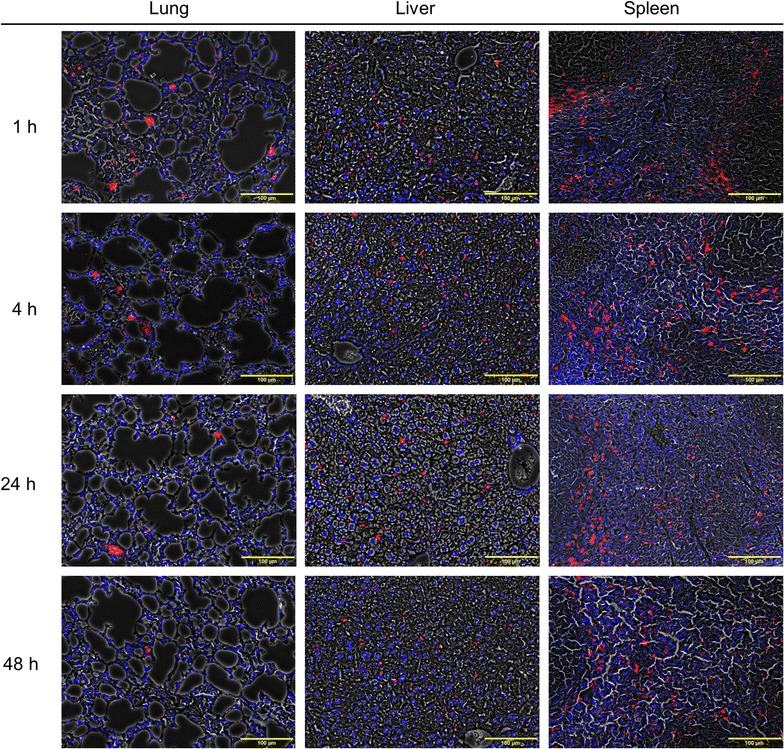
Fluorescence microscopy overlaid phase contrast images of lungs, liver and spleen. Fluorescence images of VivoTag 680XL PEG-PCL micelles are shown in red (CY5) while nuclei are stained with (DAPI) using 4′,6-diamidino-2-phenylindole (*scale bar* 100 µm). Organs were harvested at 1, 4, 24 and 48 h

**Fig. 10:**
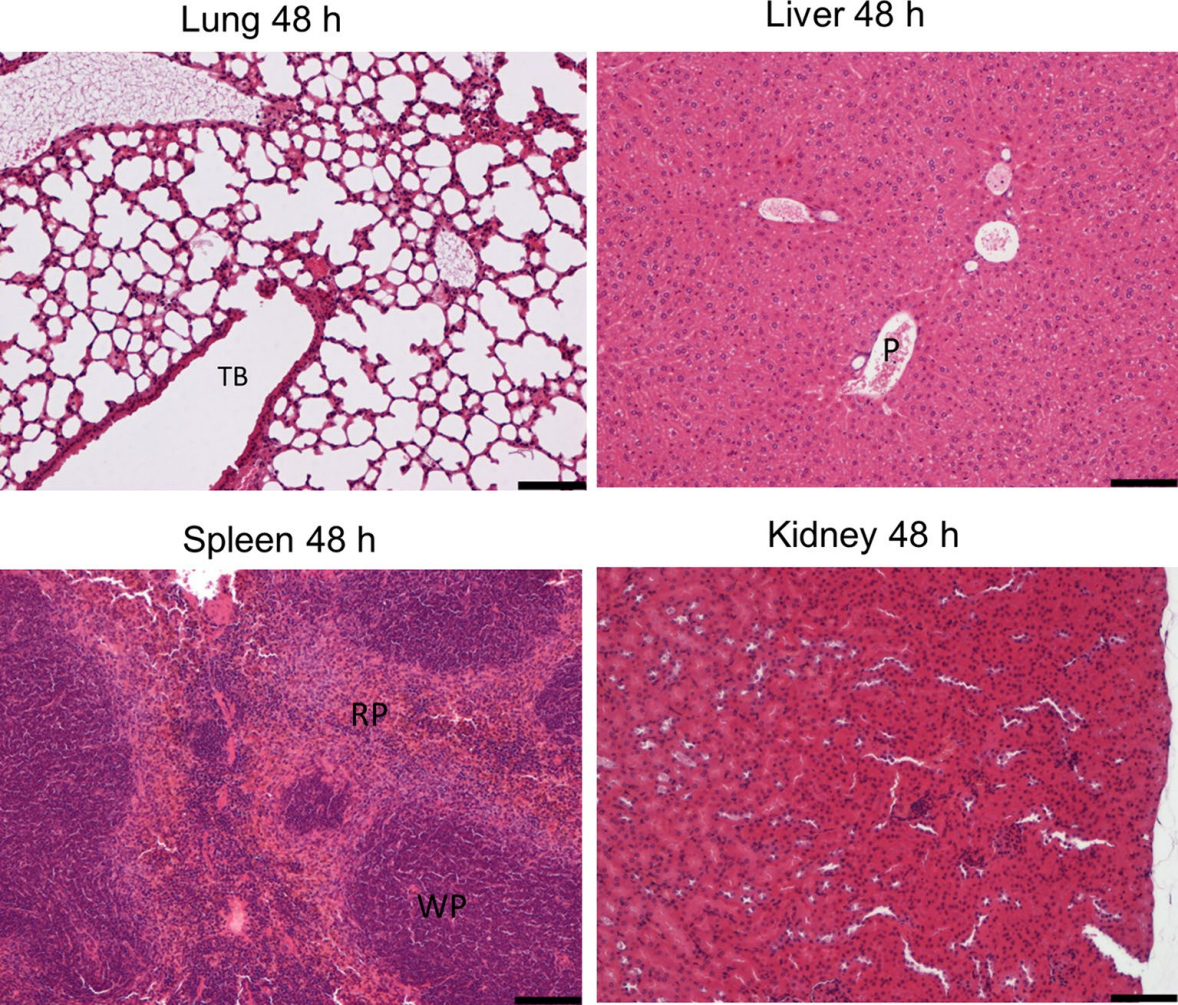
H&E staining of lungs, liver, spleen and kidney at 48 h post injection. *TB* terminal bronchiole; *WP* white pulp; *RP* red pulp; *P* portal areas. *Scale bar* 100 µm

Microscopic analysis showed the presence of micelles and agglomerates of PEG-PCL micelles in the lungs, liver, spleen and kidneys as shown in Fig. 9 and Additional file 1: Figure S4. Fluorescence microscopy with phase contrast showed tissue structure in addition to a red signal for VivoTag 680XL-labeled PEG-PCL micelles and a blue signal from DAPI stained nuclei. At early investigation time points (1 and 4 h), micelles are clearly present in the lungs as shown in Fig. 9. Moreover, single micelles or smaller aggregates of micelles are found in septal capillaries and as small endocytosed clusters in alveolar/interstitial macrophages. Notably, at 24 h post injection, relatively larger aggregates of micelles appear in larger arterioles. At 48 h post injection, very few micelles were observed in the lungs. To a higher extent, we noticed micelle presence in the liver. Histological investigation shows micelle association with sinusoidal Kupffer cells. There is no remarkable change in the amount of micelles in the liver over time. Spleen is another targeted organ of PEG-PCL micelles. The micelles were observed mainly in association with macrophages, most commonly in the marginal zone. Slight signs of clearance of micelles in the spleen and liver were observed at 48 h. Very few micelles were observed in the kidneys (Fig. S4). After Hematoxylin-eosin (H&E) staining (Fig. 10), histopathological examination was performed on the target organs to determine the organ toxicity of micelles. No evidence of toxic effect on either ion tubules or glomeruli was observed in the kidneys during the 48 h. Neither glomerular hyperemia nor glomerular hemorrhage was observed in the kidneys during the time the mice were studied. In the lungs, the alveolar areas retain normal structure at all post treatment time points. There was no sign of increased cellularity in the pulmonary septa and no macrophage accumulation in any of the mice, nor any sign of inflammation. Further, in the spleen, the white pulp (WP) areas showed normal structure and organization, while the hematopoietic red pulp (RP) areas display a relatively active extra medullary hematopoiesis as expected in young animals. Moreover, the livers retain a normal structure with a homogenous hepatocyte nuclear size. The relationship between central veins (CV) and portal areas (P) is normal. Similar observations in all organs at different time points were found (data not shown).

## Discussion

A multifunctional nanocarrier system with well-defined physico-chemical properties for anticancer drug delivery and multimodal imaging purposes was developed. In the current investigation we studied the loading capacity of busulphan into PEG-PCL micelles as a model for therapeutic anticancer drug and the control release of busulphan from micelles, the encapsulation of SPION as contrast agent to enhance MR imaging and the chemical conjugation of an NIR fluorescent molecule for multi-modal imaging capacities. Moreover, we have also investigated the cellular toxicity and organ toxicity in vivo.

The classical O/W solvent evaporation emulsion method was used to prepare PEG-PCL micelles in the presence of PVA as a stabilizer. The evaporation of the organic solvent leads to solidification of micelles [35, 36] resulting in a spherical shape and no aggregations. This method is particularly suitable for the microencapsulation of lipophilic drugs that can either be dispersed or dissolved in a volatile solvent. In this process, dichloromethane and chloroform are the most commonly used water-immiscible solvents as dispersed phase for water-insoluble polymer and drug, whereas PVA and other surfactants are widely used as emulsifiers to obtain emulsion. The micelles have an average particle size of 212 nm, but with a broad size distribution (σ ~ 30%). The broad size distribution results from the formation of micelles of amphiphilic copolymer (PEG-PCL), where the clustering of the hydrophobic tail is driven by thermodynamic equilibrium, and great freedom is allowed in the possible morphology formation. Therefore, the fast formation of a polymer shell around the oil droplets (DCM in this work) facilitates the encapsulation of water-insoluble molecules; however, the control of size distribution is weak and hence results in a broad size range. A negative zeta potential of PEG-PCL has been found, which is attributed to the negatively charged state of the PCL block in the copolymer. The high encapsulation efficiency implies that PEG-PCL micelles are suitable for busulphan delivery, due to the good affinity between busulphan and the hydrophobic core of PEG-PCL copolymer. In this investigation, we have shown a sustained release of busulphan from SPION-PEG-PCL micelles in PBS buffer solution over a period of 10 h with a burst release of approximately 22% in the first hour release pattern. The burst is attributed to diffusion of the drug located at the interface between the micelle core and the surface into the dissolution medium, and to the hydrophobicity of the drug carrier [37]. In the present study, the burst effect was controlled by increasing the initial concentration of the monomer (105 mM of ε-caprolactone) during the polymerization reaction to produce a long hydrophobic PCL chain, which reduces the initial penetration of water into the copolymer, and hence suppresses the burst release. The performance of the current drug delivery system shows much improvement regarding drug loading quantity and release profile comparing with previous reported busulphan delivery using PCL-PEG micelles [38].

The properties of SPION-PEG-PCL micelles as contrast agent were evaluated by examining the phantoms of these micelles under MRI. The *T*_*2*_ *-weighted MRI images of phantoms showed a decrease in the MR signal intensity at different TEs compared with controls. Since SPIONs can produce spin–spin relaxation effects and cause local field inhomogeneity, MRI appears predominantly dark due to shortening of the relaxation time by iron oxide. Furthermore, when the concentration of iron oxide increases, the extent of signal decrease is enhanced; i.e., much darker for a higher concentration than for a lower one. The relaxivity *r*_*2*_ * measures the change in spin–spin relaxation rate (1/*T*_*2*_ *) per unit concentration of iron oxide (mM^−1^ s^−1^). SPION-PEG-PCL micelles show a relatively high relaxation rate. As a result, the *r*_*2*_ * for the SPION-PEG-PCL micelles is significantly larger than those previously reported or commercial contrast agents such as SPION-dextran particles (30–50 Fe mM^−1^ s^−1^) [39]. The magnitude of *r*_2_ reflects the ability of materials to produce local inhomogeneity in the magnetic field, which will be affected by many factors such as particle size and state of dispersion. Our result suggests that the close-packed SPIONs in polymer micelles might contribute to the high relaxivity, and also illustrates that the formulation of SPION-PEG-PCL micelles has potential to be used as contrast agent for MRI applications.

We demonstrated the intracellular uptake of PEG-PCL micelles in a time-dependent manner. The cellular interaction with the micelles is influenced by particle size, shape, and surface charge [40–42]. Cell internalization is expected to be through phagocytosis, endocytosis and receptor mediated endocytosis as the size is below 200 nm. We also noticed that PEG-PCL micelles showed non-cytotoxicity at different concentrations in HL60 cells, which implied that the PEG-PCL micelles are safe for biomedical applications.

VivoTag 680XL is an NIR fluorescence dye with high quantum yield and high photo-stability, which allows detection of low-abundance biological structures in deep tissues with good sensitivity. VivoTag 680XL dye molecules can be attached to proteins/micelles and micro-vehicles at high molar ratios without significant self-quenching, resulting in brighter conjugates and low detection limits. The long emission wavelength of VivoTag 680XL makes it suitable for in vivo biodistribution study since there is little interference from absorption by hemoglobin (absorption peak at 550–600 nm).

In vivo biodistribution investigation revealed that after intravenous administration, the majority of PEG-PCL micelles distributed to the liver and spleen. A high amount of micelles were captured in the lungs immediately after injection followed by rapid clearance. At 1 h, the rapid distribution of PEG-PCL micelles into the lungs, liver and spleen was most probably due to the sequestration effect by the mononuclear phagocyte system (MPS). The MPS is mainly composed of resident macrophages and plays an important role in maintaining homeostasis [43]. The macrophages are found in all the connective tissues including the liver (Kupffer cells), spleen, lymph nodes, lungs and central nervous system (microglia). The uptake of PEG-PCL micelles in the lungs, liver and spleen was confirmed by histological analysis. At 1 h, micelles were found in the lungs as single particles in septal capillaries or as small endocytosed clusters in alveolar/interstitial macrophages. In the liver, micelles were associated with sinusoidal Kupffer cells, while in the spleen micelles were also observed to be affiliated with macrophages, mostly in the marginal zone. In our previous investigation of the biodistribution of carbon 11 labelled busulphan in the cynomolgus monkey, we have shown that free busulphan rapidly distributed into the liver and lungs within 1 h after iv injection. The uptake of busulphan in the lungs was 25–45% of that found in the liver [44]. At later time points (4 and 24 h), PEG-PCL micelles which were resident in the lungs were cleared by macrophages and then further accumulated in the spleen and liver. Micelle size is the major factor for micelle circulation, extravasation through vasculature leakage, macrophage uptake and renal clearance upon intravenous administration [45]. In the liver, the vascular fenestrations in non-continuous endothelia are between 50 and 100 nm, which leads to liver accumulation of micelles larger than 50 nm [45, 46]. Splenic filtration causes the retention of micelles larger than 200 nm, due to the slits between inter-endothelial cells being 200–500 nm in size [45, 47]. According to the TEM micrograph, the average diameter of our PEG-PCL micelles is 212 nm (σ ~ 30%). Therefore, the PEG-PCL micelles circulating in the blood were accumulated in the liver and spleen based on the endothelial filtration cut-off size range in those organs. At 48 h, there was a slight sign of clearance of micelles in the spleen and liver, which indicates that a part of the PEG-PCL micelles was degraded.

Nano-sized micelles have emerged as suitable delivery vehicles for anti-cancer drugs and tumor imaging contrast agents due to the enhanced permeability and retention (EPR) effect, in which fenestrated blood vessels in solid tumors lead to a high extent-accumulation of long-circulating particles/micelles [45, 48]. Once micelles are injected intravenously, protein coronas are formed on the surface of the circulating micelles facilitating the binding to receptors on the surface of phagocytes, which is followed by internalizing and fusing with lysosomes [49]. Ethylene glycol units on PEG-PCL micelles together with plasma water form a hydrating layer on the surface of the micelles, which prohibits protein adsorption and clearance by MPS [45, 50]. The surface charge of micelles also plays an important role in protein adsorption and therefore affects the biodistribution of micelles. Highly positively charged micelles are rapidly cleared from circulation while neutral and lightly negatively charged micelles have been reported as having significantly prolonged blood circulation [51]. Zeta potential measurement showed that the PEG-PCL micelles used in this study have a negative charge of approximately −2.8 mV at neutral and/or physiological pH. Hence, the PEG unit on the PEG-PCL copolymer together with the negatively-charged surface of the PEG-PCL micelles has a positive effect on particle circulation. A fluorescence signal was detectable in the femurs at 4, 24 and 48 h, suggesting that PEG-PCL micelles might also distribute to the bone marrow. The fluorescence imaging method used in the present study only revealed the biodistribution pattern of PEG-PCL micelles as delivery carriers for SPIONs and busulphan. When multiple components (for example drugs/contrast agents) are loaded into nano-carries, their distribution may differ from that of the nano-carriers due to physicochemical property change in vivo and the redistribution after released from nano-carriers [52, 53]. At the present we are, investigating and characterizing the distribution and efficacy of SPIONs and busulphan in animal models.

The preclinical biodistribution studies of these micelles in mice constitute a basis for the potential use of these carriers for lipophilic drug delivery and disease treatment. The preclinical and clinical theranostic applications of the biodegradable multifunctional micelles developed in this study could be further explored by loading other lipophilic drugs and targeting cancer models.

## Conclusions

In the present study,

- Biodegradable multifunctional nanoparticle system containing busulphan was developed.
- SPIONs were encapsulated into the PEG-PCL nanocarrier, as a contrast agent to enhance *T*_2_*-weighted MR imaging.
- Fluorescence-labeled PEG-PCL nanocarriers allowed for real-time investigation of the dynamic biodistribution in vivo and the uptake by different organs during a 48 h period.
- No tissue impairment or toxicity was observed during the time the study was conducted.

We strongly believe that the SPION-PEG-PCL nanocarrier is a promising theranostic candidate for biomedical applications, where in vivo imaging and controlled drug delivery for therapy are necessary.

## Additional file

**Additional file 1.** Dynamic light scattering (DLS) of SPION-PEG-PCL micelles, cytotoxicity of PEG-PCL micelles, co-registration of 3D FLIT/CT imaging, and fluorescence microscopy images of kidney.
