# Supplementary material for: Biodistribution of biodegradable polymeric nano-carriers loaded with busulphan and designed for multimodal imaging
Source: J Nanobiotechnology. 2016 Dec 19;14:82. doi: 10.1186/s12951-016-0239-0 (PMC5168852; doi:10.1186/s12951-016-0239-0)

**Supporting Information**

### Biodistribution of Biodegradable Polymeric Nano-carriers Loaded with Busulphan and Designed for Multimodal Imaging

**Heba Asem^1, 2*^, Ying Zhao^2, 3*^, Fei Ye^2^, Åsa Barrefelt^2^, Manuchehr Abedi-Valugerdi^2^, Ramy El-Sayed^2^, Ibrahim El-Serafi^2^, Khalid M. Abu-Salah^4^, Jörg Hamm^5^, Mamoun Muhammed^1^, Moustapha Hassan^2,3^**

^1^ Division of Functional Materials (FNM), Department of Materials and Nanophysics, Royal Institute of Technology (KTH), SE-164 40 Stockholm, Sweden.

^2^ Division of Experimental Cancer Medicine (ECM), Department of Laboratory Medicine (LABMED), Karolinska Institutet (KI), SE-141 86 Stockholm, Sweden.

^3^ Clinical Research Center (KFC), Karolinska University Hospital Huddinge, SE-141 86, Stockholm, Sweden.

^4^ King Abdullah International Medical Research Center, Department of Nanomedicine, King Abdulaziz Medical City, PO Box 22490, Riyadh 11426

^5^ PerkinElmer, 68 Elm St., Hopkinton, MA 01748, USA

* Equal contribution.

**Corresponding author:**

Moustapha Hassan, Clinical Research Center (KFC), Karolinska University Hospital Huddinge, SE-141 86 Stockholm, Sweden. Telephone: +46-8-58583862.

E-mail: Moustapha.Hassan@ki.se

**Running header: Multimodal Imaging and Biodistribution of Biodegradable Polymeric Micelles**

**Figure S1. Dynamic light scattering (DLS) of SPION-PEG-PCL micelles dispersed in DI water.**

The hydrodynamic diameter is represented by differential volume, intensity, or number, respectively. The surface charge of SPION-PEG-PCL micelles is also measured and it showed a negative zeta potential of ca. -2.8 mV at neutral pH.

**
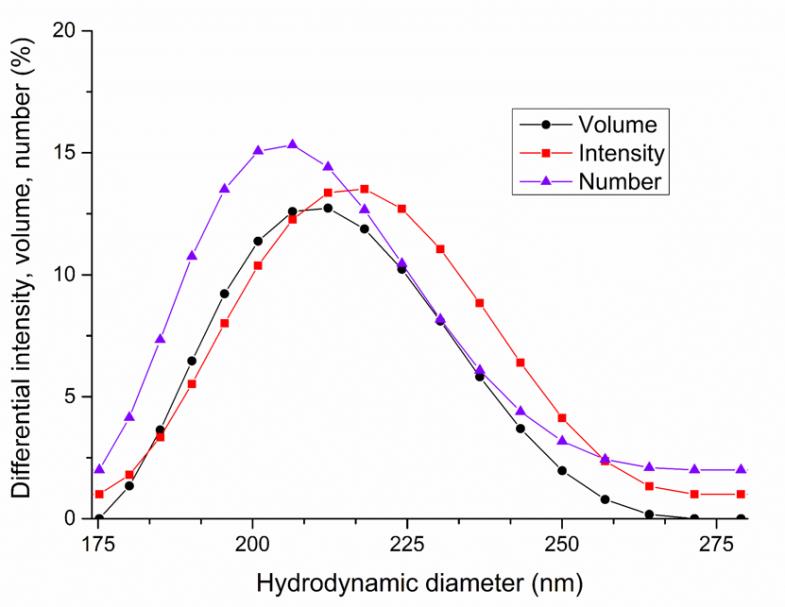
**

**Figure S2. Cytotoxicity of PEG-PCL in HL60 cell line.**

The cell viability was determined after 48 h incubation with PEG-PCL micelles in HL60 cells at different concentrations. The cell viability was determined using MTT assay and was calculated as the percentage of living cells after treatment with micelles to that of the non-treated cells.
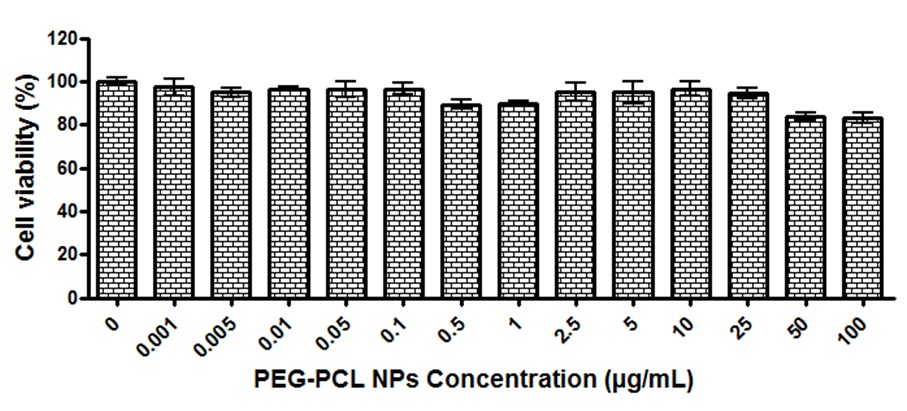


**Figure S3. 3D Fluorescence imaging tomography-CT imaging co-registration**

The 3D FLIT imaging with trans-illumination in a dorsal position sequence was set up and images were acquired at excitation 675 nm and emission 720 nm. The mouse in the MIS was then transferred to the Quantum FX-µCT and subjected to a fast, low dose CT scan with a field of view (FOV) at 60 mm and 17 second scan-time. The slice views show the images of coronal, sagittal and transversal plane of balb/c mice at 1, 4, 24 and 48 h post i.v. administration. At 1 h after administration signal was observed in lungs, spleen and liver. During the time of the study, the signal was redistributed mainly, to liver and spleen.

**
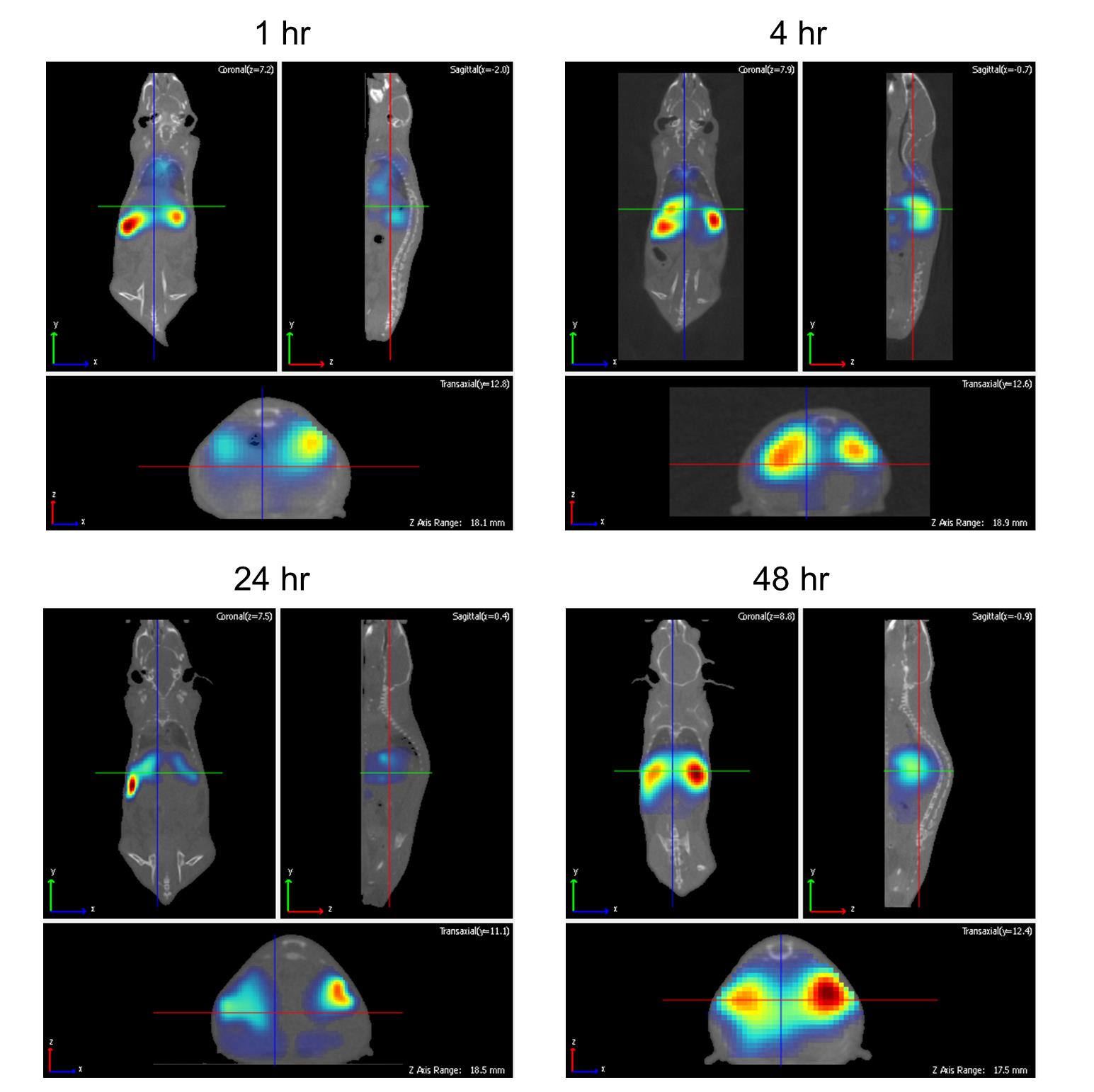
**

**Figure S4**. **Fluorescence microscopy overlaid phase contrast images of kidney.**

Fluorescence images of VivoTag 680XL tagged PEG-PCL micelles are shown in red while nuclei are stained with (DAPI) using 4',6-diamidino-2-phenylindole (scale bar=100 µm). Very few micelles were observed in the kidneys. Neither glomerular hyperemia nor glomerular hemorrhage was observed in the kidneys during the study time.


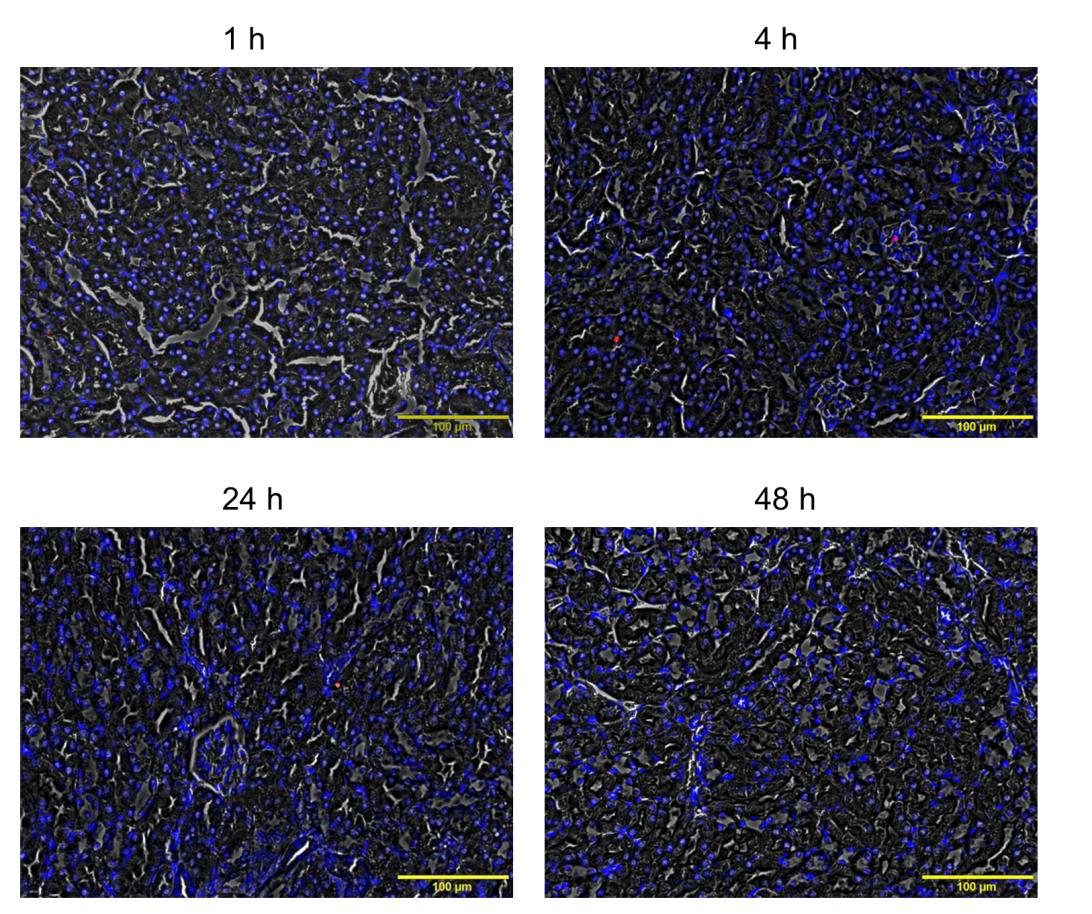

Supplement: Supplementary file 1 — Additional file 1. Dynamic light scattering (DLS) of SPION-PEG-PCL micelles, cytotoxicity of PEG-PCL micelles, co-registration of 3D FLIT/CT imaging, and fluorescence microscopy images of kidney. [file 12951_2016_239_MOESM1_ESM.docx]
